# Digital teaching with interactive case presentations of ENT diseases – discussion of utilisation and motivation of students

**DOI:** 10.3205/zma001393

**Published:** 2020-12-03

**Authors:** Veronika Vielsmeier, Steffen Auerswald, Jörg Marienhagen, Stephanie Keil, Nico Müller

**Affiliations:** 1 Universitätsklinikum Regensburg, Klinik und Poliklinik für Hals-Nasen-Ohrenheilkunde, Regensburg, Germany; 2 Universität Augsburg, Studiengang Humanmedizin, Augsburg, Germany; 3 Universität Regensburg, Fakultät für Medizin, Studiendekanat, Regensburg, Germany

**Keywords:** digital teaching, practical relevance, case presentation, motivation, Covid-19

## Abstract

**Introduction:** Due to the circumstances of the Covid-19 pandemic, the teaching during the block internship at the Department for Otorhinolaryngology was switched to digital learning. Various online courses were created and the utilisation by the students was analyzed.

**Material & methods:** Examination videos, surgical images and videos were created and live lectures were held. In addition, patient cases of common otorhinolaryngological diseases were reconstructed on an interactive platform. A total of 16 cases were offered in weekly rotation. These cases are provided with gap texts, open and selection questions, links and videos and thematically appropriate digression offers. The time-consuming creation was carried out as a HTML 5 learning package with the authoring program Exelearning 2.5. Each case was to be evaluated separately after being worked on by the students.

**Results:** The direct feedback and the evaluation results of the students on the internship and case presentations were consistently positive. However, on average only 50.72% of the registered students took part in the weekly video meetings. In the course of the semester, the willingness to participate decreased. In addition, the willingness to evaluate the patient cases was low.

**Discussion: **With the case presentation tool, concrete patient examples can be well presented, especially when patient contact is not possible (especially in an ENT clinic due to violation of distance and hygienic rules). Even though the evaluations were positive in terms of content, the frequency of utilisation and also the motivation for feedback seems disappointing. This seems to be associated above all with an increasing return to everyday life after the end of the lockdown.

## Zusammenfassung

**Einleitung: **Aufgrund der Umstände der Covid-19-Pandemie wurde der Unterricht während des Blockpraktikums der Klinik und Poliklinik für Hals-Nasen-Ohrenheilkunde auf digitales Lernen umgestellt. Diverse Online-Lehrangebote wurden erstellt und die Nutzung der Studierenden analysiert.

**Material & Methoden: **Es wurden Untersuchungsvideos, Operationsbilder und -videos erstellt sowie Live-Vorlesungen abgehalten. Darüber hinaus wurden Patientenfälle von häufigen HNO-Erkrankungen rekonstruiert auf einer interaktiven Plattform angeboten. Insgesamt wurden 16 Fälle im wöchentlichen Wechsel angeboten, diese sind mit Lückentexten, offenen und Auswahl-Fragen, weiterführenden Links und Videos und thematisch passenden Exkurs-Angeboten versehen. Die zeitaufwendige Erstellung erfolgte als HTML 5 Lernpaket mit dem Autorenprogramm Exelearning 2.5. Jeder Fall sollte nach Bearbeitung durch die Studierenden separat evaluiert werden.

**Ergebnisse: **Die direkten Rückmeldungen und die Evaluationsergebnisse der Studierenden zum Praktikum und zu den Fallbearbeitungen waren durchweg positiv. An den wöchentlichen Videomeetings haben jedoch zum Beispiel im Schnitt nur 50,72% der angemeldeten Studierenden teilgenommen. Im Laufe des Semesters nahm die Teilnahmebereitschaft ab. Zudem war die Bereitschaft die Patientenfälle zu evaluieren gering.

**Diskussion: **Mit dem Tool der Fallvorstellung können konkrete Patientenbeispiele, insbesondere bei nicht möglichem Patientenkontakt (vor allem in der HNO wegen Verletzung der Abstandsregeln), gut dargestellt werden. Auch wenn die Evaluationen inhaltlich positiv waren, erscheint uns die Nutzungshäufigkeit und auch Motivation zum Feedback enttäuschend. Dies scheint vor allem mit zunehmender Rückkehr zum Alltagsgeschehen nach dem Ende des Lockdowns assoziiert zu sein.

## Introduction

The Covid-19 pandemic has had a major impact on teaching in medical schools [1] and new digital tools had to be developed [2]. The teaching of the Department of Otorhinolaryngology has been completely transformed into digital teaching. Reasons for this were the distance rules to patients which could not be observed, because during examinations mouth and nose protection can naturally not be worn and close contact is necessary, also in endoscopic techniques. This leads to an increased risk of infection for students and patients [3], [4]. In addition, some of the students were deployed to Covid-19 wards or testing facilities during the pandemic and thus carried an increased risk of infection. Furthermore, the university pointed out that, as far as possible, attendance at lectures should be avoided. 

The challenge is that practical training is very limited and patient contact, which is essential in human medicine studies, is not possible. However, practical relevance is considered indispensable for good training and education of medical doctors [5], [6]. Therefore the processing of patient cases as close to reality as possible should be a decisive aspect of the training. 

However, earlier studies, such as those by Daubenfeld et al. also showed that new teaching concepts or digital learning do not necessarily perform better than traditional concepts [7], [8], [9]. Therefore, the aim of this study was to evaluate novel teaching concepts and to register their utilisation. 

## Material & methods

In order to switch the content of the teaching at the Department of Otorhinolaryngology to teaching without presence, the main lecture was held as a synchronous online lecture (via zoom). The contents of the block practical course were completely converted to digital distance teaching, i.e. examination videos, surgical pictures and teaching videos were created and disposable instruments (nasal speculum, mouth spatula, ear speculum) were handed out for home use. 

Due to the missing possibility of presence teaching and patient contact, the creation of patient cases, which were provided on a learning platform, was also carried out. In addition, there is the problem that during the otorhinolaryngological examinations the mouth-nose protection is not worn by the patients and the distance cannot be kept during the examination. Therefore, we developed a total of 16 virtual cases of common ENT diseases, of which two cases per week were offered in weekly rotation, for example epistaxis, acute tonsillitis or laryngeal carcinoma. These are provided with cloze texts, open and selection questions, further links and videos. In some cases, thematically appropriate digression offers were added, e.g. internal comorbidities in epistaxis. The time-consuming creation was done alternately by medical staff as HTML 5 learning package with the authoring program Exelearning 2.5 (source: [https://exelearning.net/en/]). 

The students were divided into eight groups for one week each (12-15 participants each) similar to the face-to-face classes and were informed about the weekly schedule with daily tasks by individualized mail. Two cases were to be worked on and evaluated each Thursday. On Fridays, participants were expected to attend a 45-60-minutes video meeting to discuss content and answer questions. 

To enable clinical practice with patient contact and examination techniques, students were offered a short clinical traineeship (over five days). The evaluation of the newly developed interactive patient case management was conducted using the survey software EvaSys.

## Results

138 students in the sixth clinical semester were registered to participate in the lecture and the block internship. 

**Figure 1:**
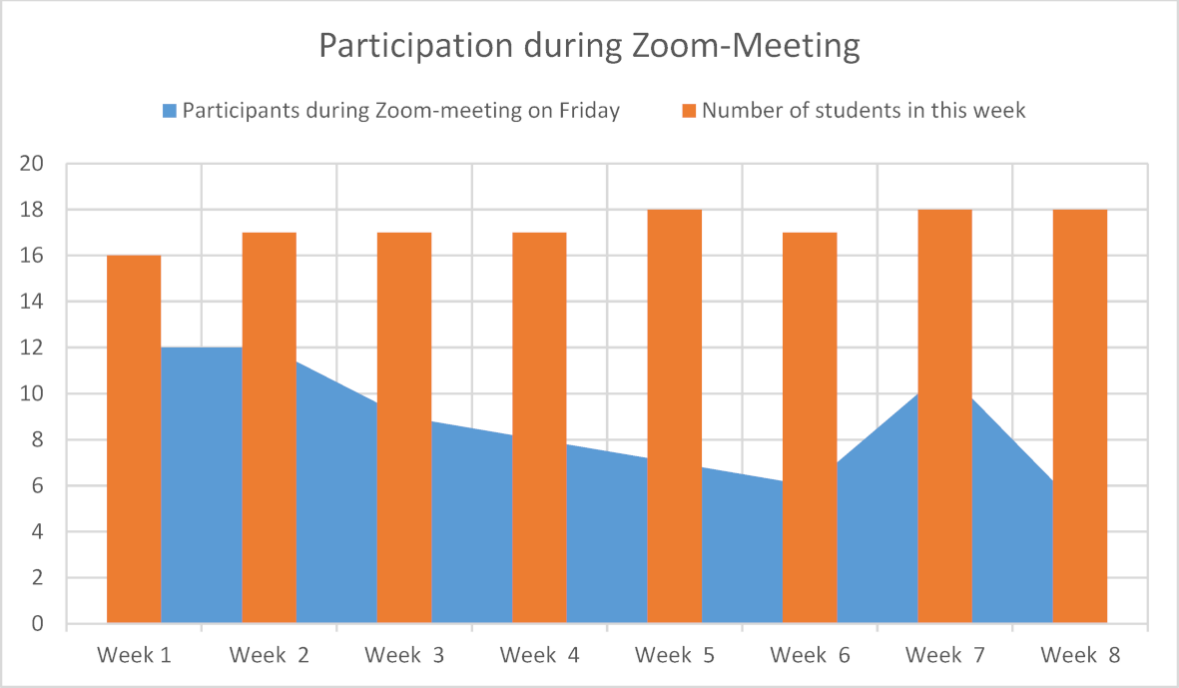
Number of participants during the weekly Zoom-meeting (absolute)

**Figure 2:**
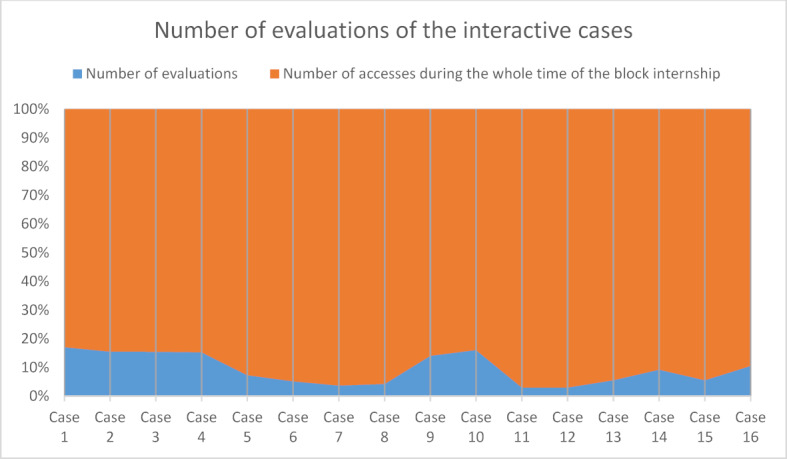
Percentage of evaluations of the interactive cases by students

The disposable instruments offered were collected (or sent out as required) by 65 of 138 students (47.10%). The weekly video meeting was attended by 70 of 138 students (50.72%), although there was a clear decline in participation over time (see figure 1 (Fig. 1)). 2 of the 138 students (1.45%) took advantage of the short clinical traineeship offered. In addition, it was noticeable that despite repeated oral and written requests (because it was a new tool) only a small number of students evaluated the newly developed case presentations (see figure 2 (Fig. 2)). 

## Discussion

The short-term changeover to the new teaching situation in the context of the Covid-19 pandemic was a major challenge for teachers in the study of human medicine. Due to the limited opportunities for attendance, alternative courses had to be created quickly and at great expense. The Department of Otorhinolaryngology offered, among other things, an online block internship, which was supplemented in particular by reconstructed cases with interactive processing options. 

However, it turned out that the use by the students was disappointing and still has potential improvement. Only 1.45% of the students took advantage of the offer of a short attendance time to learn practical exercises. The offered free disposable instruments for the examination exercises at home were used by only half of the students, same at a 45-60 minutes video meeting to discuss the contents. It is particularly noticeable that participation over the period of the eight-week block internship showed a declining tendency with a weekly different group. However, since the group size and the semester were the same, the change of group cannot have a decisive influence on the decline in participation. 

Since case-based learning is considered promising [10], we supplemented our course offerings with patient cases. The evaluation for this was very satisfactory in terms of content, but participation in the evaluation fell far short of expectations despite several indications. This seems disappointing, especially under the aspect that a new teaching tool should be evaluated and optimized. 

Direct contact with teachers and motivation are known to be important reasons for learning success [5]. It can be speculated whether the limited motivation due to decreasing interest in the subject, the time consumed by competing study tasks such as exams, or the increasing return to everyday life could be associated with corresponding diversionary activities after the lockdown. 

It seems that only direct contact with students and their presence can ensure sufficient motivation to use the courses, even for students who are less interested in the subject or discipline. In order to ensure that every student deals with basic content of otorhinolaryngology, we therefore believe that compulsory attendance classes in small groups with corresponding attendance and learning controls are essential. In the future, the goal has to be to control the processing and use of online courses in a digital form, which complement the classroom teaching.

## Acknowledgements

We would like to thank Mrs. Birgit Scheungrab for her administrative support in drawing up the plans and communicating with the students.

## Competing interests

The authors declare that they have no competing interests.
